# Clinical Perspectives on Stroke Prevention in Patients with Non-valvular Atrial Fibrillation in the Philippines

**DOI:** 10.7759/cureus.90214

**Published:** 2025-08-16

**Authors:** Rodney Jimenez, Alisa Bernan, Federick C Cheng, Herminigildo H Gan, Maria Victoria Garcia, Helen Ong-Garcia, Jermaine Lim

**Affiliations:** 1 Cardiology, University of the East Ramon Magsaysay Memorial Medical Center, Quezon City, PHL; 2 Cardiology, St. Luke’s Medical Center - Global City, Taguig City, PHL; 3 Cardiology, National Research Council of the Philippines, Philippine Heart Association, Davao City, PHL; 4 Cardiology, Accreditation Committee of Specialty Boards on Adult Cardiology, Philippine Heart Association, Pasig, PHL; 5 Diagnostic Division, HBC-Heart and Vascular Institute, St. Luke's Medical Center, Quezon City, PHL; 6 Neurology, Jose R. Reyes Memorial Medical Center, City of Manila, PHL; 7 Medicine, University of Santo Tomas, City of Manila, PHL; 8 Stress Laboratory, St. Luke's Medical Center - Global City, Taguig City, PHL; 9 Cardiac Rehabilitation Unit, Chinese General Hospital, City of Manila, PHL; 10 Cardiology, Cardiac Rehabilitation Society of the Philippines, Quezon City, PHL; 11 Medical Affairs, Viatris, Taguig City, PHL

**Keywords:** apixaban, atrial fibrillation, gaps in healthcare, healthcare access, nonvalvular atrial fibrillation, oral anticoagulants, patient adherence, philippines, stroke prevention

## Abstract

Stroke prevention in patients with non-valvular atrial fibrillation (NVAF) is a public health priority in the Philippines. This paper aims to assess the existing gaps in stroke prevention and management for patients with NVAF in the Philippines. It focuses on identifying stroke risk, evaluating barriers to care, and ensuring treatment adherence. Barriers such as financial constraints, lack of awareness, and difficulties in accessing healthcare facilities hinder timely diagnosis and proper management. Despite the availability of stroke-ready hospitals, gaps in stroke care infrastructure, including a shortage of specialized healthcare professionals, continue to affect patient outcomes and delay intervention. To improve stroke prevention in patients with NVAF, a comprehensive strategy is needed. This approach should focus on enhancing healthcare infrastructure, expanding public and healthcare provider education, and addressing disparities in care. Furthermore, ensuring access to effective pharmacological options is essential. Non-vitamin K oral anticoagulants, which may be more suitable for resource-limited settings, offer advantages such as reduced risk of bleeding and minimal monitoring requirements. A patient-centric approach that prioritizes adherence and access to pharmacological treatments can help reduce the stroke burden and improve long-term outcomes for patients with NVAF in the Philippines.

## Introduction and background

Atrial fibrillation (AF) is the most common sustained arrhythmia globally, contributing significantly to patient morbidity and healthcare burden [1]. Over the past decade, the global prevalence of AF has markedly increased, with cases rising from 33.5 million in 2010 to 59 million in 2019 [2]. Many individuals with AF are unaware of their condition due to the lack of symptoms and limited access to screening programs [3]. In addition, public understanding of AF is low; only 48% of individuals globally can accurately identify the condition and its associated risks [4]. Although no study has precisely quantified the number of undiagnosed AF cases in the Philippines, this combination of asymptomatic presentation, insufficient screening, and poor awareness strongly suggests that the true burden is significantly underestimated. In 2017 and 2018, 10,563 PhilHealth Insurance claims for hospitalization in the Philippines were attributed to AF, reflecting a notable impact of this condition on the healthcare system [5]. Risk factors include increasing age, hypertension, smoking, diabetes mellitus, heart failure, obesity, illicit drug use, obstructive sleep apnea, excessive alcohol intake, and physical inactivity [6-9]. Importantly, AF remains a key modifiable risk factor for ischemic stroke [10]. While a study conducted in Cebu City, Philippines, assessed the prevalence of metabolic syndrome among adult Filipinos with thyroid disease, it did not specifically evaluate hyperthyroidism as a stroke risk factor [11]. Interestingly, a national survey in the Philippines reported a thyroid dysfunction prevalence of 8.53%, with subclinical hyperthyroidism being the predominant form at 5.33% [12]. Although hyperthyroidism has not been well studied in local stroke data, international studies have established a link between hyperthyroidism and an increased risk of ischemic stroke, highlighting the relevance of endocrine factors in stroke pathophysiology [13,14].

Non-valvular AF (NVAF) is the more prevalent form of AF that significantly increases the risk of ischemic stroke by four- to five-fold [15,16]. NVAF manifests in 5% to 10% of patients with mild heart failure (symptoms with exertion), 10% to 26% with moderate heart failure (symptoms with minimal activity), and up to 50% of those with advanced heart failure (symptoms even at rest) [17,18]. Furthermore, NVAF is more prevalent among older individuals and is associated not only with more severe outcomes and greater disability following a stroke event, but also with an increased risk of developing dementia [17,19,20]. Ischemic stroke, which accounts for 70% of stroke cases, remains a leading cause of disability and mortality globally, especially in low- and middle-income countries like the Philippines [21-23]. In 2020, stroke was the third leading cause of death in the Philippines, accounting for 10.4% of the reported fatalities [24,25].

Despite the young population, stroke consistently ranked among the top five cases of disability in the country from 2009 to 2019 [22,24,25]. Due to limited epidemiological data, the actual prevalence of AF-related stroke remains uncertain [21,26], with reported estimates ranging from 0.4% to 6.0% of the population [22]. Stroke care in the country faces significant challenges, including inadequate diagnostic tools, a shortage of specialists, and limited access to treatment, with only 49 stroke-ready hospitals available. This shortage hampers timely intervention and comprehensive patient management, affecting overall outcomes [8,21,27]. There is a scarcity of neurosurgeons and neurologists, with a ratio of 1 per 218,000 Filipinos, who are heavily concentrated in urban areas [8,21]. Additionally, emergency medical services are underdeveloped, and overcrowding of public hospitals leads to delays in care [9,21]. Limited community awareness about stroke and inadequate government insurance coverage further hinders timely diagnosis and treatment [9]. These issues are compounded by disparities in healthcare access between urban and rural areas, financial barriers, and limited rehabilitation facilities [8,28]. The migration of healthcare professionals (HCPs) seeking better compensation further exacerbates the problem [29].

Preventing stroke in patients with NVAF is crucial, given the significant risks associated with the condition. Oral anticoagulants (OACs), including vitamin K antagonists (VKAs, e.g., warfarin) or non-VKA direct oral anticoagulants (DOACs, e.g., apixaban, rivaroxaban, edoxaban, dabigatran), are essential in mitigating this risk [30]. Several guidelines recommend DOACs as the preferred alternative for the treatment of patients with NVAF [31-33]. However, patient adherence to anticoagulation therapy poses a significant challenge, particularly in resource-constrained settings like the Philippines [34]. Non-adherence may result in poor health outcomes, increased risk of ischemic and hemorrhagic complications, and higher healthcare costs [30,34,35].

This review highlights the gaps in stroke prevention for patients with NVAF in the Philippines, focusing on the patient journey, barriers to care, and challenges to treatment adherence. It offers recommendations to improve access to healthcare and optimize patient outcomes.

## Review

Patient journey

Identification of Patients At-Risk for Stroke

Identifying patients at high risk for stroke in the Philippines involves assessing a broad range of factors predisposing them to this serious health concern (Table 1). In patients with AF who were hospitalized for stroke in a tertiary hospital in Cebu City, Philippines from 2015 to 2022, mortality was linked to specific factors, including the type of AF, age ≥70 years, red cell distribution width, neutrophil-to-lymphocyte ratio, platelet count, and low-density lipoprotein cholesterol levels [36]. The other key risk factors include female gender, comorbidities like cardiovascular diseases, including hypertension, AF, congestive heart failure, diabetes, and those with a previous history of stroke, embolism, intracranial hemorrhage, or a transient ischemic attack [37,38]. Patients with a family history of stroke [39], or genetic sequence variations like PITX2 and ZFHX3 located in chromosomes 4q25 and 16q22, respectively, require vigilant monitoring [40,41].

**Table 1:** Temporal course of neurological patient management in the Philippines: From early risk detection to long-term follow-up AF, atrial fibrillation; CT, computed tomography; DOAC, direct oral anticoagulant; ECG, electrocardiogram; MRI, magnetic resonance imaging; VKA, vitamin K antagonist

Stage	Duration	Key Actions	Intervention Points
Risk identification (ongoing)	Continuous for high-risk individuals or annual for general screening	Identify risk factors (e.g., age >65 years, hypertension, diabetes, atrial fibrillation); use CHA₂DS₂-VA and HAS-BLED scoring for stratification	Start lifestyle counselling and community awareness initiatives
Screening and initial presentation	1–4 weeks depending on access to healthcare facilities	Screen for AF using ECG, Holter monitoring, or smart devices; assess symptoms (e.g., palpitations, fatigue, stroke precursors); perform CHA₂DS₂-VA scoring	Conduct public awareness for symptom identification; promote affordable, accessible diagnostic services
Diagnosis	1–3 days	Confirm AF with 12-lead ECG, electrocardiography, and biomarkers; use imaging techniques (e.g., transcranial Doppler) for embolic risks	Rapid diagnostic confirmation
Treatment initiation	1–2 weeks post-diagnosis	Start anticoagulants (e.g., DOACs or VKAs) tailored to individual needs; implement multidisciplinary care with neurologists and cardiologists	Ensure affordability and availability of medications; address patient education and adherence barriers
Acute management	1–3 days post-event	Emergency care, thrombolysis, imaging (CT/MRI), and stabilization; collaborate with multidisciplinary teams for acute care	Create rapid hospital access pathways; follow clinical stroke management protocols
Rehabilitation and follow-up	1–6 months post-treatment	Enroll in physical therapy and rehabilitation programs; monitor anticoagulant therapy; adjust treatment as needed	Involve families in recovery planning; provide periodic reassessment and patient support
Long-term management	Lifelong (6+ months post initial diagnosis)	Continue monitoring and managing modifiable risk factors; use digital tools and personalized multidisciplinary care	Enhance access to specialized care and medication; focus on prevention of recurrent stroke

Despite limited access to advanced diagnostics, recent local efforts such as barangay-level blood pressure screening and AF detection campaigns using handheld electrocardiogram (ECG) devices in rural health units demonstrate potential for early risk identification [42-44]. Expanding such initiatives could significantly improve early detection rates, especially in underdiagnosed patients in remote regions.

In geographically isolated and disadvantaged areas, community health workers have begun playing a critical role in identifying patients at risk of stroke through structured questionnaires and symptom-based screening. Training community health workers in AF risk recognition can facilitate timely referral and bridge the gap in rural healthcare delivery.

Biomarkers like increased levels of troponins, natriuretic peptides, D-dimer, von Willebrand factor, soluble E-selectin, P-selectin, interleukin-6, and C-reactive protein also suggest a high risk of stroke [40]. Risk stratification tools like the CHA2DS2-VAscore consider factors like a history of congestive heart failure/left ventricular dysfunction, hypertension, age ≥75 years, age 65-74 years, previous stroke/transient ischemic attack/thromboembolism, and vascular disease [45,46]. Each factor is assigned 1 point except for previous stroke/transient ischemic attack/thromboembolism and age ≥75 years, which are assigned 2 points. A score of 2 or more identifies patients at high risk for an embolic stroke. Anticoagulation decisions must also account for bleeding risks using the HAS-BLED score, which evaluates hypertension, abnormal renal/liver function, stroke history, bleeding history or predisposition, labile international normalized ratio, age, and concomitant use of drugs or alcohol. A HAS-BLED score of 3 or higher signifies increased risk of bleeding, emphasizing careful consideration of treatment strategies [45,46].

Lifestyle factors like tobacco and excessive alcohol consumption, unhealthy diet, sedentary behavior, and stress also significantly contribute to stroke risk [47]. Moreover, marginalized communities facing socioeconomic disparities often lack access to preventive healthcare, exacerbating their susceptibility to stroke [48,49].

In certain Filipino communities, stroke symptoms are often attributed to supernatural or non-medical causes, leading to delays in seeking medical care. Culturally sensitive education campaigns are vital to counter misinformation and promote timely diagnosis and treatment. The Philippines Neurological Association’s PNA1DB-stroke ready registry has underscored the limited detection of AF among elderly stroke patients, pointing to a potential underdiagnosis of AF in patients aged ≥70 years, and highlighting the need for targeted screening in this high-risk group [38]. Integrating such registry insights and risk identification models may improve predictive accuracy for the local population. Addressing AF screening gaps in the Philippines aligns with broader global initiatives to reduce cardiovascular mortality under WHO’s Universal Health Coverage goals. Local improvements in community-based screening and training of primary care providers can serve as scalable models for other low- and middle-income countries facing similar disparities.

Recognizing these multifaceted risk factors and implementing targeted screening initiatives can effectively identify patients at high risk and enable timely interventions to reduce the burden of stroke in the Philippines.

Screening, Clinical Presentation, and Diagnosis

Identifying AF is crucial for preventing strokes, especially when symptoms are subtle or absent. As a modifiable risk factor, timely diagnosis and management of AF can significantly improve clinical outcomes, leading to better prognosis, reduced risk of stroke and heart failure, and more effective overall treatment. Early identification empowers HCPs to deliver personalized treatment plans, track disease progression more accurately, optimize therapeutic strategies, and actively involve patients in their own care, thereby improving their long-term outcomes and quality of life. The AF-CARE approach, as proposed in the 2024 ESC guidelines, will make the management of AF both holistic and patient centered, where C is for comorbidity and risk factor management, A is for avoid stroke and thromboembolism, R is for reduce symptoms by rate and rhythm control, and E is for evaluation and dynamic reassessment [7].

The symptoms of AF are diverse. Contrary to popular belief, palpitations are less common than non-specific complaints like fatigue, anxiety, and at times, depression. Patients may only become aware of their rhythm abnormality due to associated comorbidities. Older individuals may occasionally present symptoms of heart failure. Elevated heart rates during AF episodes can result in chronic heart failure, often requiring hospitalization.

Early diagnosis of AF is vital for reducing its associated morbidity, mortality, and economic burden. However, detecting the condition remains challenging due to the low diagnostic yield of conventional methods. The 12-lead ECG remains the gold standard of AF diagnosis [50]. Diagnosis can also rely on clinical assessments, supported by laboratory tests and, potentially, biomarkers. Following an initial clinical assessment, the HCPs may request additional tests to establish a definitive diagnosis.

Barriers such as limited training and awareness among healthcare professionals in rural settings may contribute to the underutilization of risk stratification tools like CHA2DS2-VASc and HAS-BLED, potentially affecting anticoagulation decisions in patients with AF [37]. Without standardized risk assessment, patients at high risk for stroke may remain untreated or improperly anticoagulated, contributing to poorer outcomes and increased healthcare burden, an issue particularly relevant for the rural health system globally.

Diagnostic techniques like Holter monitoring, echocardiography, and carotid function assessment may be deployed following initial clinical assessment. Holter monitoring is selective based on need, while the transcranial Doppler bubble test can detect or rule out patent foramen ovale with a sensitivity of 97% and specificity of 93%, making it useful for detecting embolic strokes in patients with paroxysmal AF, thereby enhancing the screening process [51]. However, there are challenges like time and cost constraints. While standard ECGs, Holter monitors, Loop recorders, and even ECG apps on smartwatches show promise in detecting AF, their widespread use is often restricted due to time, costs, and accessibility [52].

Recent research has introduced the ACL score as a novel risk stratification tool to improve the prediction of AF in patients with cryptogenic stroke. Developed using a combination of clinical factors, baseline ECG findings, short-term rhythm monitoring, and ECG data, the ACL score demonstrated superior predictive performance compared to the commonly used CHA2DS2-VASc score and was comparable to the C2HEST score. By accurately identifying patients’ risk for device-detected AF, the ACL score enables more selective and cost-effective use of implantable cardiac monitors. This tailored approach can support earlier detection and intervention, ultimately contributing to better secondary stroke prevention and optimized resource utilization [53].

Advancements offered with artificial intelligence (AI)-based ECG software can help accurately predict the risk of developing AF in patients with sinus rhythm, incorporating additional biomarkers like troponin into risk assessment protocols [54]. Smartphone, smartwatch, and other devices are increasingly recommended for screening of AF, alongside traditional methods like ECG and echocardiography [55].

CHA2DS2-VA and HAS-BLED scores are often used to assess thromboembolic and bleeding risks. However, these scores may not apply universally, highlighting the need for a personalized approach. When there is a discrepancy between these scores, the balance between bleeding and clotting risks must be carefully weighed. High CHA2DS2-VA and low HAS-BLED scores may warrant DOACs, while equal scores prompt consideration. In cases where the CHA2DS2-VA score is low, the risk of stroke is minimal, so anticoagulation is usually not needed [56]. However, if anticoagulation is required for another reason, a high HAS-BLED score (≥3) calls for caution, close monitoring steps to reduce risks of bleeding, such as managing hypertension or avoiding nonsteroidal anti-inflammatory drugs (NSAIDs) [57,58]. Dose adjustments in such cases depend on renal function, age, and body weight rather than directly on the CHA2DS2-VA or HAS-BLED scores [59]. These scores guide the decision to start anticoagulation but not the dosing, highlighting the need for individualized therapy and regular reassessment. 

Numerous biomarkers linked to the extent of tissue damage caused by infarction have been recognized, such as S-100B, matrix metalloproteinase, interleukin-6, tumor necrosis factor-alpha, intercellular adhesion molecule-1, and glutamate. These indicators hold potential in predicting the clinical outlook for patients undergoing ischemic strokes [60,61]. Additionally, other blood-based biomarkers, including natriuretic peptides, stress response markers like copeptin and cortisol, inflammation markers such as procalcitonin and mannose-binding lectin, and markers associated with atherogenesis like adipocyte fatty acid-binding protein, have emerged as promising indicators for predicting the prognosis of ischemic strokes [61].

In the Philippines, the factors linked to higher mortality rates were assessed using ECG, complete blood count, platelet count, renal and liver function tests, and lipid profile. Other suggested assessments include CHA2DS2-VA score, Modified Rankin Scale score, and National Institutes of Health Stroke Scale score for assessment of stroke severity [36].

Stroke prevention in patients with AF, particularly NVAF, hinges on the timely initiation of anticoagulation therapy. Oral anticoagulants, including DOACs, significantly reduce the risk of stroke. The CHA2DS2-VA score supports risk-based anticoagulation decisions, especially in high-risk NVAF patients. For patients with a high thromboembolic risk, anticoagulation therapy is indispensable.

Approach to Patient Care

Managing stroke in patients with AF often involves a collaborative effort among the HCPs. The process typically begins when patients or their caregivers recognize symptoms and seek medical attention. Upon arrival at the healthcare facility, emergency medical personnel, including paramedics and emergency department staff, play a pivotal role in assessing the patient and stabilizing their condition. Initial interventions may include oxygen therapy and intravenous medications to address acute needs. A crucial principle in stroke management is ‘Time is Brain’, which implies that patients must be brought to the hospital within 4.5 hours of symptom onset to allow timely administration of thrombolytics and improve outcomes (Table 2) [62,63]. Among patients with ischemic stroke, it is imperative to perform a computed tomography scan first to determine the type of stroke (ischemic vs. hemorrhagic), as thrombolytic therapy is contraindicated in hemorrhagic stroke. This imaging step ensures appropriate and safe management. Once the patient is stabilized, a multidisciplinary team comprising neurologists, cardiologists, radiologists, and other specialists collaborates to design a tailored treatment plan. This plan may include anticoagulation therapy, interventional procedures, and rehabilitation therapies to prevent future complications and optimize recovery.

**Table 2:** Stepwise algorithm for the initial management of ischemic stroke

Stage	Description/Key Actions
Recognize stroke symptoms	Sudden onset of facial drooping, arm weakness, or speech difficulty
	Other symptoms may include vision disturbances or loss of coordination
Immediate action	Active emergency response by calling emergency services
	Ensure transport to the hospital within 4.5 hours of symptom onset
Initial hospital assessment	Evaluate vital signs and neurological status
	Perform stroke severity assessment
	Perform brain computed tomography to determine the type of stroke
Treatment pathways	Within 4.5 hours: Administer intravenous thrombolytics
	Beyond 4.5 hours or with large vessel occlusion: Evaluate eligibility for endovascular thrombectomy
	If not eligible for reperfusion: Provide supportive care
	Blood pressure must be optimally controlled before initiating thrombolytic therapy
Post-treatment monitoring	Monitor for bleeding and neurological recovery
	Plan early rehabilitation and implement stroke prevention measures
	Stabilize blood pressure, heart rate, and rhythm before intervention

Aside from thrombolytic and anticoagulant therapies, effective management of blood pressure is also critical in the acute and chronic phases of care. Tight blood pressure control reduces the risk of recurrent stroke and helps prevent further cardiovascular complications [64]. Guidelines recommend initiating or adjusting antihypertensive agents post-stroke based on individual targets, comorbidities, and tolerability [65-67].

Symptom management in patients with AF involves both rate and rhythm control strategies. Rate control is achieved using beta-blockers, calcium channel blockers, or digoxin to maintain an appropriate ventricular rate, particularly in patients who are asymptomatic or minimally symptomatic [68,69]. Rhythm control, on the other hand, may be considered in patients with persistent symptoms or heart failure and includes the use of antiarrhythmic drugs or electrical cardioversion. The choice between rate and rhythm control should be individualized, with shared decision-making between the patient and physician.

If a stroke patient has been treated with thrombolysis (e.g., using alteplase or tenecteplase), antiplatelet and anticoagulant medications should generally be withheld for the first 24 hours. This precaution helps reduce the risk of bleeding, particularly intracranial hemorrhage, which is a known complication after thrombolytic therapy [70].

While DOACs offer advantages over traditional vitamin K antagonists, such as reduced need for monitoring and a lower risk of intracranial hemorrhage, the potential for bleeding complications cannot be ignored. The HAS-BLED score is a valuable tool to assess the risk of bleeding and guide clinical decisions. Concomitant use of drugs (aspirin, heparin, thrombolytic agents, selective serotonin reuptake inhibitors, serotonin-norepinephrine reuptake inhibitors, and NSAIDs) affecting hemostasis increases the risk of bleeding [71]. Patients should be made aware of the signs and symptoms of blood loss and instructed to report them immediately or seek emergency care. DOAC therapy should be discontinued in patients with active pathological hemorrhage. For severe bleeding associated with dabigatran, idarucizumab, the only approved antidote, can be used to reverse its effects [72]. For apixaban, rivaroxaban, and edoxaban, and in cases where specific antidotes are unavailable, supportive measures, careful monitoring, and hemodynamic stabilization are essential to ensure patient safety. Once bleeding is controlled and clearance from HCPs is obtained, resuming anticoagulation therapy is often appropriate to continue protecting against thromboembolic events. Effective communication among HCPs, including emergency personnel, neurologists, and cardiologists, is critical to ensure seamless transitions between acute and chronic phases of care.

Ultimately, the goal of managing stroke in patients with AF is to optimize outcomes by integrating anticoagulation therapy, managing the risk of bleeding, and providing comprehensive patient education. A collaborative and multidisciplinary approach combined with personalized treatment plans enables healthcare teams to address the unique needs of each patient, ensuring long-term safety and efficacy. HCPs, often in the specialty of neurology, in the Philippines tackle the issue of stroke through evidence-backed and cost-effective strategies outlined in the clinical practice guidelines provided by the Stroke Society of the Philippines [73,74]. Neurologists play a vital role in the diagnosis, treatment, and overall management of stroke cases across the country. By collaborating with emergency physicians, internists, radiologists, cardiologists, rehabilitation specialists, and nurses, they ensure a multidisciplinary approach to patient outcomes and quality of life.

Access to Treatment

Access to treatment of stroke in low- and middle-income countries, including the Philippines, is a significant challenge due to disease burden, barriers to adequate care, and high treatment costs. In a public tertiary hospital in the Philippines between 2017 to 2018, healthcare expenses were linked to factors such as the type of stroke, Glasgow Coma Scale score, surgical procedures, intravenous thrombolysis, infections, the duration of hospitalization, and the need for mechanical ventilation [75]. Around half a million Filipinos could be affected by stroke, with medical expenses ranging from $350 million to $1.2 billion [23]. While the Philippine Health Insurance Corporation (PhilHealth) reimburses professional and healthcare institution fees ($1300 (Php 76,000) for ischemic stroke and $1368 (Php 80,000) for hemorrhagic stroke), additional expenses for treatments like thrombolysis, including emergency cranial CT scans, laboratory tests, medication, emergency room fees, and physician charges, can be significant [9,76]. In private hospitals, thrombolysis cost can range from $2,733 to $4,573 (Php 136,688 to Php 228,678), while in government hospitals, it can range from $65 to $718 (Php 3,239 to Php 35,903) [9]. The median hospitalization cost for strokes in a tertiary public hospital could be $329.64 (Php 17,141.50) [75]. These expenses could strain finances, particularly for families with lower incomes. Additionally, the cost of rehabilitation after stroke poses further challenges, as current PhilHealth packages has low coverage for these expenses, leaving patients to bear the financial burden. Limited coverage for rehabilitation services, financed primarily out of pocket, creates accessibility issues for lower-income groups.

Adherence to the Treatment

The global landscape presents a myriad of challenges to treatment adherence, spanning from socioeconomic disparities to cultural beliefs and healthcare access. Stigma around certain conditions can hinder individuals from seeking or adhering to treatment regimens. Logistical barriers such as the availability of healthcare facilities, transportation, as well as medication costs further complicate adherence efforts. Complex treatment protocols and a lack of patient education can also contribute to non-adherence. Ensuring that patients with stroke stick to their prescribed medications is crucial for preventing further health complications. In the Philippines, adherence to treatment is not well captured and reported. There is a lack of information on how well patients adhere to their medication regimens in this country. The Prospective Urban Rural Epidemiology (PURE) study in South Asia reported an 80% lack of medication adherence due to underutilization of healthcare services, reduced access to curative care, low household wealth index, and illiteracy [77]. Failing to take medications as directed can result in recurrence, increased disability, and even death among survivors.

Barriers to medication adherence include financial constraint, negative beliefs about medication, multiple doses, forgetfulness, perception of illness, medical beliefs, long-term treatment schedule, past negative experiences with healthcare services, difficulty accessing health care, inadequate community care, poor patient-physician relationship, and medication side effects [78-82]. Propagation of fake news and the magnification of adverse effects of medications, such as DOACs, on social media further exacerbate patient hesitation and mistrust. Addressing these barriers involves overcoming financial constraints, educating patients and their caregivers about the benefits and risks of DOACs (including addressing the fear of bleeding), simplifying treatment plans, and providing reminders for medication intake. Involving family members in the treatment process and improving communication between patients and HCPs are also vital strategies to improve medication adherence. Educating patients and caregivers with the help of dos and don’ts and raising awareness through the distribution of informative materials can further bolster efforts to promote adherence. Many patients with stroke or AF present with fears that doctors should address. Ensuring affordable, reliable medications and training physicians to address patient concerns effectively are also crucial.

Furthermore, there is an emergent need to adhere to the local and international guidelines for improving stroke outcome. Local medical societies, like the Stroke Society of the Philippines, the Philippine Society of Vascular Medicine, and the Philippine Heart Association, regularly adapt international guidelines to fit the local context.

Pharmacological treatments for stroke prevention in patients with NVAF

**Figure 1:**
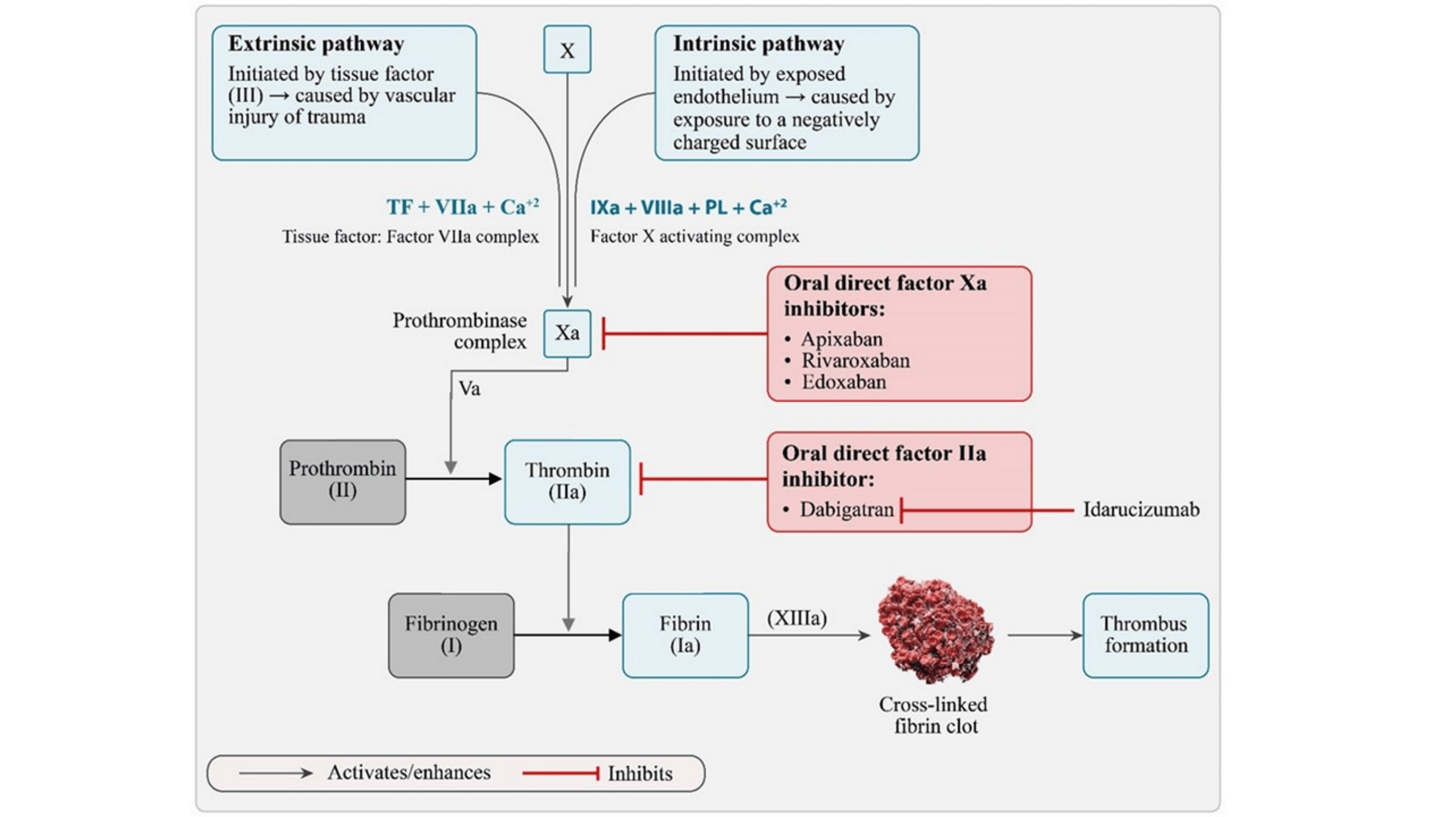
Mechanism of action of DOACs DOACs: direct oral anticoagulants; TF: tissue factor; PL: phospholipid Figure 1 is the author's own creation using data from references [87,88]

Pharmacological interventions play a pivotal role in preventing stroke in patients with NVAF. While various therapeutic options such as VKAs and DOACs are available for managing stroke in patients with AF, VKAs are hindered by their narrow therapeutic range, requiring frequent monitoring and dosage adjustments [83]. Additionally, VKAs can also induce vitamin K deficiency by inhibiting the enzyme vitamin K epoxide reductase, potentially impacting bone health [84]. To overcome these limitations, DOACs have emerged as a promising alternative for the management of NVAF. DOACs offer several advantages over VKAs, including a lower bleeding risk profile, reduced need for routine coagulation monitoring, simplified fixed-dose regimens, quicker onset of action, and fewer food and drug interactions [85]. Patients with moderate to severe mitral stenosis and those with prosthetic mechanical heart valves represent populations that are ineligible for DOAC therapy; for these groups, VKAs remain the recommended treatment option [86]. Currently, four types of DOACs (apixaban, rivaroxaban, edoxaban, and dabigatran) are available for the treatment of patients with NVAF [87,88]. These agents are indicated for lowering the risk of stroke in patients with NVAF and share a similar mechanism of action. Apixaban, rivaroxaban, and edoxaban are highly competitive, selective, and potent direct inhibitors that bind to the active site of factor Xa, inhibiting the conversion of prothrombin to thrombin, the final enzyme in the coagulation cascade. By inhibiting factor Xa, they halt the progression of the cascade and prevent the formation of a clot. Dabigatran, in contrast, functions as a factor IIa inhibitor (thrombin), binding with high affinity to the active site of thrombin and blocking its ability to convert fibrinogen to fibrin, thereby also preventing clot formation (Table 3; Figure 1) [87-92].

**Table 3:** Comparative pharmacodynamics and pharmacokinetics of DOACs ACS: acute coronary syndrome; ASA: acetyl salicylic acid; BID: twice-daily; DVT: deep vein thrombosis; h: hour; FDA: Food and Drug Administration; L: liter; MI: myocardial infarction; DOACs: non-vitamin K direct oral anticoagulants; NVAF: non-valvular atrial fibrillation; OD: once-daily; PE: pulmonary embolism; SE: systemic embolism; VTE: venous thromboembolic events; Vd: distribution volume Source: [89-92]

	Apixaban [89]	Rivaroxaban [90]	Edoxaban [91]	Dabigatran [92]
Type	Small molecule	Small molecule	Small molecule	Small molecule
Approved indications	Reduce stroke and SE in patients with NVAF; prevention of VTE after elective hip or knee replacement surgery; treatment of DVT or PE, or prevention of their recurrence	Prevention of CV death, MI, and stent thrombosis in patients after an ACS in combination with ASA alone or with ASA + thienopyridines clopidogrel or ticlopidine; prevention of stroke, MI, CV death, acute limb ischemia, and mortality in patients with CAD or PAD in combination with ASA; prevention of stroke and SE in patients with NVAF; prevention of VTE in patients undergoing hip or knee replacement surgery Treatment of DVT or PE, or prevention of their recurrence	Reduce stroke and SE in NVAF; treatment of DVT or PE or prevention of their recurrence	Prevention of stroke, SE, and reduction of vascular mortality in patients with AF; prevention of acute DVT and/or PE and prevention of related death; treatment of DVT and/or PE and prevention of related death
Target	Factor Xa	Factor Xa	Factor Xa	Factor IIa
Prodrug	No	No	No	Yes
Route of administration	Orally	Orally	Orally	Orally
Dose and frequency	5 and 2.5 mg, BID	2.5, 10, 15 and 20 mg, OD or BID depending on indication	30 and 60 mg, OD	75, 110 OD, and 150 mg, OD or BID depending on indication
Absorption	Proximal small bowel + gastric	Proximal small bowel + gastric	Proximal small bowel	Lower stomach + duodenum
Bioavailability (%)	50	66	62	3 to 7
Vd (L)	21	50	107	50–70
Protein binding (%)	87	>90	55	35
Time to peak concentration (h)	1–4	2–4	1–2	1–2
Half-life (h)	12	5–9	10–14	12–17
Elimination	Renal and hepatic	Renal and hepatic	Renal and hepatic	Renal

Evidence from randomized controlled studies supports the use of DOACs as preferred anticoagulation therapy for patients with NVAF [93,94]. In patients with AF who were not candidates for warfarin, apixaban, as compared with aspirin, reduced the rate of stroke or systemic embolism (SE) by 55% without increasing the risk of major bleeding [95]. Meta-analysis of pivotal phase 3 studies has shown that DOACs as compared with warfarin offer superior prevention of SE or cerebral ischemia, with a reduction in hemorrhagic stroke and intracranial hemorrhage. Furthermore, DOACs also lowered severe major bleeding events than warfarin [96]. Another meta-analysis of seven phase 3 studies demonstrated that DOAC treatment was associated with lower mortality rates compared with VKAs in patients with NVAF [97].

Primary stroke prevention in patients with NVAF without a history of stroke or transient ischemic attack but having other risk factors plays a crucial role in reducing cardiovascular events. The goal of anticoagulation therapy in this population is to prevent the first stroke while balancing bleeding risks. DOACs, including apixaban, rivaroxaban, dabigatran, and edoxaban, have shown superior or comparable efficacy to VKAs in stroke prevention while significantly reducing intracranial hemorrhage risks. Rivaroxaban (hazard ratio 0.65; 95% confidence interval (CI): 0.45-0.90) and dabigatran 150 mg twice-daily (risk ratio 0.60; 95% CI: 0.45-0.078) demonstrated notable effectiveness compared to VKAs in clinical studies [98,99]. Real-world studies further highlight that rivaroxaban reduced stroke risk by 19% and mortality by 24% compared to warfarin [100], while apixaban and dabigatran yielded similar benefits [101]. For patients seeking greater stroke risk reduction, rivaroxaban and dabigatran may be preferred. Conversely, apixaban and edoxaban may offer a safer profile for patients with previous life-threatening bleeding events [102]. Personalized treatment, guided by clinical judgment and patient preferences, remains vital for optimizing outcomes.

Numerous publications further support the preference for DOAC over VKA therapy. For example, data from studies including patients with AF receiving different anticoagulant therapies for two years revealed that renal function deterioration was significantly lower in those treated with DOACs vs. VKAs [103]. Additionally, the Taiwan National Health Insurance Research database, followed up for 2.4 years, showed higher risks of fractures and diabetes mellitus associated with VKA therapy [104,105].

DOACs offer a favorable balance between reducing thromboembolic events and minimizing bleeding risks, establishing them as superior to VKAs [106]. However, in frail or older patients, caution is advised due to potentially higher bleeding risks. The FRAIL-AF study showed that frail patients who switched to DOAC from VKA therapy had an increased risk of bleeding, though thromboembolic events and mortality rates were similar to those noted with VKA therapy. This bleeding may indicate underlying oncological pathology, making it a potential marker for early detection and prognosis [107].

In the management of thromboembolic events, restarting DOACs benefits patients compared to withholding them, despite an increased risk of bleeding. Restarting DOACs is associated with reduced mortality risk. Some factors that may lead to withholding anticoagulation include previous bleeding, frailty, and overall high bleeding risk. In patients with NVAF at high risk of gastrointestinal (GI) bleeding, apixaban, dabigatran, and rivaroxaban were linked to lower stroke risks compared with warfarin, with apixaban and dabigatran also showing lower risks of major bleeding compared with warfarin. In a Korean dataset of AF patients with prior GI bleeding, DOACs demonstrated significantly lower risks across various primary and secondary outcomes compared with warfarin [108].

Numerous epidemiological studies have linked AF to an increased risk of cognitive impairment and dementia [109-112], and such patients often do not receive adequate treatment. AF contributes to cognitive decline through multiple pathophysiological mechanisms, including silent cerebral infarction, cerebral hypoperfusion due to irregular heart rhythm, microbleeds, and systemic inflammation, all of which can lead to neuronal injury and degeneration [20]. When comparing non-treatment to DOACs and warfarin, DOACs show lower risks of ischemic stroke and major bleeding, while warfarin is associated with a higher risk of intracranial hemorrhage. Approximately three-quarters of AF patients fall into the pre-frail or frail category, with frail patients exhibiting increased risks of death, stroke, and bleeding, yet they are often undertreated with anticoagulants. Warfarin is not the preferred anticoagulant for frail patients, as all DOACs demonstrate lower rates of stroke, bleeding, and death compared with warfarin in this group. In patients considered unstable, including those with labile international normalized ratio, frequent bleeding or thromboembolic events, poor adherence to medication, high risk of falls, significant comorbidities, or complex drug interactions, apixaban shows reduced rates of adverse events compared with warfarin [113-115].

In patients with rheumatic heart disease (moderate to severe mitral stenosis), DOACs were associated with higher mortality rates compared with VKAs according to the INVICTUS study. Therefore, VKA remains the preferred treatment for patients in this group [116]. Similarly, in patients with mechanical prosthetic heart valves, another subgroup of valvular AF, DOACs are not recommended. The RE-ALIGN study evaluating higher doses of dabigatran in this population was terminated early due to increased thromboembolic events and bleeding complications [117]. A study with apixaban also showed unfavorable outcomes [118], reinforcing that VKAs remain the standard of care in these patients. Conversely, in patients with bioprosthetic heart valves, emerging evidence suggests that DOACs may be a safe alternative to VKAs, particularly in those without other contraindications [119,120]. 

In scenarios requiring dose reduction or complex management, the dose reduction schemes for DOACs are well-defined and align with recommended guidelines [33]. The European Heart Rhythm Association (EHRA) practical guide provides comprehensive tables of different drug interactions with DOACs, highlighting combinations to avoid and emphasizing factors like dosing, age, renal function, and other practical considerations [33]. Managing patients on DOACs can be challenging, especially in scenarios like concurrent antiplatelet therapy or conditions like deep vein thrombosis, pulmonary embolism, and patients with compromised renal function or undergoing dialysis. It is essential to carefully consider these factors and consult resources like the EHRA practical guide to ensure optimal treatment outcomes for patients [33].

Upon analyzing bleeding-related hospitalizations in AF patients using amiodarone or flecainide/sotalol alongside apixaban or rivaroxaban, it was observed that the incidence of bleeding was notably higher in the amiodarone group. This was notable especially among patients receiving rivaroxaban or those with known risk factors for hemorrhagic complications of anticoagulant treatment. Furthermore, a study conducted in the USA analyzing safety and efficacy in NVAF patients across different body mass index (BMI) categories (underweight, overweight, obesity grade 1-2, obesity grade 3) showed beneficial outcomes with DOACs in all BMI groups [121]. Similarly, in the Korean data set stratified by BMI, no significant interaction was observed between BMI and the efficacy and safety of DOACs compared to warfarin [122].

In the ARISTOPHANES study that included an obese patient cohort, apixaban demonstrated preferable effectiveness and safety compared to rivaroxaban, with the latter showing a higher risk of bleeding than dabigatran [123]. Among cancer patients in the same study, apixaban showed lower risks of stroke/SE and major bleeding compared with warfarin, while dabigatran and rivaroxaban had risks similar to warfarin [123]. Data from the Taiwan registry indicated that cancer patients treated with DOACs had better outcomes in various cardiovascular and bleeding events compared to those treated with warfarin [124].

In AF patients with chronic kidney disease (CKD), DOACs, especially apixaban and edoxaban, demonstrated superior efficacy and safety over warfarin. Apixaban was particularly associated with a lower risk of major bleeding in patients with advanced CKD [125]. In general AF patient populations, apixaban was associated with a lower risk of GI bleeding and similar rates of ischemic stroke/SE, intracranial hemorrhage, and all-cause mortality compared to dabigatran, edoxaban, and rivaroxaban. The 2018 European Heart Rhythm Association guidelines advise against using DOACs in AF patients with severe CKD or those undergoing dialysis [126,127]. Patients with additional risk factors should be closely monitored, particularly if on dabigatran. Fluctuations in renal function due to acute illness should prompt re-evaluation, with the re-check interval determined using the CrCl/10 formula when renal function is impaired (CrCl ≤60 mL/min) [33]. Among DOACs, apixaban may be considered for use in AF patients with a CrCl <15 mL/min [128,129].

Real-world studies also provide valuable insights into treatment effectiveness, safety, patient preferences, and long-term consequences of treatments, complementing the findings of clinical studies. Beyond controlled studies, understanding how DOACs perform in real-world scenarios is crucial for treatment decisions. A recent study comparing adverse events between real-world AF patients anticoagulated with VKAs from the Murcia AF Project and the AMADEUS trial cohort found significant heterogeneity between the two populations. This variability corresponded to a higher risk of major bleeding, ischemic stroke, and all-cause mortality in the real-world cohort, with HR of 6.32 (95% CI, 2.84-14.03) for major bleeding, 3.56 (95% CI, 1.22-10.42) for ischemic stroke, and 5.13 (95% CI, 3.02-8.69) for all-cause mortality. Additionally, the risk of all other adverse events was elevated in real-world patients compared to those in randomized clinical trials, except for cardiovascular mortality [130]. The ARISTOPHANES study showed that apixaban demonstrated effectiveness and a more favorable safety profile than rivaroxaban and dabigatran. Dabigatran showed better efficacy than rivaroxaban but had a similar safety profile [131]. Another study utilizing the South Korean National Insurance database showed that DOACs were associated with lower risks of ischemic stroke, intracranial hemorrhage, GI bleeding, major bleeding, and the composite outcome than warfarin [132]. When comparing different DOACs, dabigatran and edoxaban exhibited lower rates of certain adverse events compared with rivaroxaban and apixaban. Apixaban and edoxaban were associated with a lower rate of GI bleeding when compared with rivaroxaban. Overall, with similar rates between each other, apixaban, dabigatran, and edoxaban were associated with a lower rate of major bleeding vs. rivaroxaban [132]. Data from the Taiwan national insurance database further supported the benefits of DOACs over warfarin in terms of reduced risks of ischemic stroke/SE, bleeding, and intracranial hemorrhage, even among high-risk subgroups and older patients ≥80 years of age [133]. A recent multinational study compared DOACs with each other, demonstrating that apixaban had a lower risk of GI bleeding and similar rates of ischemic stroke or SE, intracranial hemorrhage, and all-cause mortality compared with dabigatran, edoxaban, and rivaroxaban. The standard dose and low-dose regimen of apixaban and rivaroxaban also showed the same pattern [134]. In patients aged ≥80 years, DOACs were associated with lower risk of dementia, all-cause mortality, and ischemic stroke compared to warfarin or no DOACs [135]. A similar trend was observed in patients aged ≥90 years and in extremely high-risk groups, especially with apixaban showing benefits over warfarin and no anticoagulant treatment. These findings are particularly significant given the underrepresentation of such patient groups in clinical studies [136].

Another treatment aspect is tackling the cases with multimorbidity. The Charlson Comorbidity Index (CCI) is a measure used to identify patients with multimorbidity. Among patients without AF, the average CCI tends to rise with age, indicating increased multimorbidity. However, AF patients generally have significantly higher average CCI scores compared to those without AF. This higher level of multimorbidity (CCI score above 4) is associated with elevated risks of stroke, bleeding, and mortality. Interestingly, despite the heightened risks, there is an inverse correlation between increasing multimorbidity and the prescription of anticoagulants. In the GLORIA-AF phase 3 study involving patients with one or more stroke risk factors eligible for anticoagulation, approximately 71% had multimorbidity [137]. Among NVAF patients with multimorbidity from the ARISTOPHANES study, both apixaban and rivaroxaban were associated with lower risks of stroke/SE versus warfarin. Apixaban and dabigatran exhibited lower risks of major bleeding, while rivaroxaban posed a higher risk than warfarin. Additionally, apixaban showed a lower risk of stroke/SE compared with dabigatran, though dabigatran had a higher risk than rivaroxaban. Both apixaban and dabigatran also had lower risks of major bleeding compared to rivaroxaban [123].

Advancing AF care: tailored treatment and holistic management****

In the past 15 years, the landscape of anticoagulant therapy for AF has expanded significantly with the introduction of four new DOACs. Guidelines such as those from the American College of Chest Physicians' (CHEST) emphasize the importance of tailoring treatment based on individual patient characteristics, guiding decisions between VKAs or specific DOACs [138]. Despite anticoagulation therapy, patients with AF continue to face a notable risk, including ischemic stroke. Consequently, there is a growing focus on managing modifiable risk factors to mitigate these risks. The 2024 European Society of Cardiology (ESC) practice guidelines advocate for a comprehensive approach known as the CARE pathway. This framework focuses on managing comorbidities, preventing stroke, controlling symptoms, and ensuring continuous reassessment of treatment. Central to this approach is patient-centered, multidisciplinary care aimed at optimizing outcomes [7]. The ESC guidelines feature dedicated section on anticoagulation management in older adults and those with cognitive dysfunction, aligning with current evidence. Importantly, they highlight the limited evidence supporting anticoagulation in frail, multimorbid older adults, including those with dementia. Effective AF care extends beyond mere treatment prescriptions; it requires a holistic, patient-centered approach that addresses individual needs, simplifies treatment regimens to minimize side effects, and ensures access to multidisciplinary care. Shared decision-making is key, empowering patients through education, practical tools like apps and reminders, and tailored protocols that consider factors such as gender and socioeconomic status. Regular follow-ups by a multidisciplinary team ensure the treatment plan adapts to the patients’ changing needs. By combining personalized treatment with a collaborative, holistic management strategy, the guidelines aim to improve patient adherence and achieve better long-term outcomes in AF care [7].

Expert opinion

Expert recommendations underscore the critical need for a comprehensive and patient-centered approach to managing AF, recognizing the diverse array of factors influencing treatment decisions and outcomes. Central to this approach is the integration of local and international guidelines, with a nuanced understanding of how these recommendations apply within specific healthcare contexts. Tailoring management strategies to individual patient profiles, including age, comorbidities, and socioeconomic factors, is essential for optimizing therapeutic efficacy while minimizing potential risks.

Risk stratification tools remain foundational in treatment decisions. Routine use of CHA2DS2-VA and HAS-BLED scores supports a structured approach to balancing stroke prevention and bleeding risk. This risk-based approach guides the selection of appropriate anticoagulant agents, with a growing preference for DOACs like apixaban compared to VKA for prevention of stroke in patients with NVAF. Establishing a panel of biomarkers is also important along with genetic markers. This will strengthen diagnostic accuracy and allow for personalized treatments. Additionally, expert consensus emphasizes the importance of leveraging both traditional and emerging diagnostic modalities to facilitate early detection and characterization of AF. While ECG remains a cornerstone in AF diagnosis, the integration of innovative technologies such as smartwatch ECG apps and AI-driven predictive models offers additional insights into arrhythmia patterns and risk stratification.

To enhance the confidence of primary care providers in initiating treatment for AF, it is essential to address concerns surrounding bleeding risks. Educational programs should emphasize the effective use of risk assessment tools, such as CHA2DS2-VA and HAS-BLED, to balance thrombotic and bleeding risks. Conducting workshops, offering decision making support tools, and promoting mentorship with cardiologists can further encourage safe and timely anticoagulation at the primary care level. Clear, practical protocols tailored for primary care settings can help reduce unnecessary referrals and improve patient outcomes.

In parallel, proactive patient education and engagement are pivotal components of effective AF management, empowering individuals to make informed decisions about their treatment while fostering medication adherence and lifestyle modifications. Multidisciplinary collaboration among HCPs, including cardiologists, neurologists, and primary care physicians, facilitates coordinated care delivery and seamless transitions between acute and chronic management phases.

Moreover, ongoing medical education initiatives aimed at both clinicians and patients are essential for fostering a culture of continuous learning and empowerment within the AF care continuum. By embracing evidence-based practices, personalized medicine principles, and collaborative care models, clinicians can optimize patient outcomes while navigating the complexities of AF management in real-world clinical settings (Table 4).

**Table 4:** Key barriers with potential solutions and proposed actions AI: artificial intelligence; AF: atrial fibrillation; CV: cardiovascular; CT: computed tomography; FAST: face, arm, speech, and time; MRI: magnetic resonance imaging; TV: television

Category	Barriers	Potential Solutions	Proposed Actions
Infrastructure	Inadequate stroke-ready hospitals. Limited CT/MRI machines. Scarcity of neurologists and neurosurgeons, especially in rural areas. Overcrowding in public areas.	Expand government investment in healthcare facilities. Incentivize specialists to work in underserved areas. Strengthening telemedicine initiatives.	Increase funding for building/renovating stroke units. Introduced loan forgiveness for medical graduates serving in remote areas. Deploying mobile diagnostic units in rural regions.
Healthcare access	High out-of-pocket costs. Limited insurance coverage for thrombolysis and rehabilitation. Geographic barriers in rural areas.	Expand PhilHealth coverage to include full stroke management. Subsidize transportation costs for rural patients.	Develop policies to enhance insurance coverage. Create satellite rehabilitation units in remote areas.
Treatment	Financial constraints. Lack of awareness about medication importance. Fear of side effects. Forgetfulness.	Patient education campaigns. Simplify medication regimens Use technology (e.g. reminder through apps).	Partner with community health workers to educate patients. Distribute educational materials and set up support groups.
General awareness	Low awareness about stroke symptoms and prevention. Delayed recognition of AF, especially in asymptomatic cases Cultural beliefs and practices influencing health-seeking behavior. Spread of miscommunication and fake news through social media and community networks, contributing to misconceptions and treatment hesitancy.	Nationwide campaigns on stroke signs (e.g., FAST) and AF screening. Leverage AI and wearable devices for AF detection.	Organize public health campaigns on TV/radio. Provide free community screening events.

## Conclusions

The introduction of DOACs has transformed stroke prevention in patients with AF, particularly NVAF, by offering improved efficacy and safety over VKAs. This review uniquely highlights the Filipino healthcare context, where the implementation of DOACs is challenged by disparities in access, cost barriers, and gaps in healthcare infrastructure. By integrating clinical evidence with real-world considerations in the Philippines, this paper provides a comprehensive perspective on optimizing NVAF management. It emphasizes the importance of patient education, treatment adherence, healthcare provider collaboration, and guideline adaptation to local contexts. Furthermore, this paper sheds light on the specific needs of frail, elderly, and multimorbid Filipino patients, contributing novel insights into practical risk stratification, pharmacologic selection, and individualized care pathways. Strengthening access to DOACs and supporting a multidisciplinary, patient-centered model can reduce the burden of stroke and improve long-term outcomes for patients with NVAF in underprivileged regions of the Philippines.
